# An effectiveness-implementation hybrid trial study protocol targeting posttraumatic stress disorder and comorbidity

**DOI:** 10.1186/s13012-016-0424-4

**Published:** 2016-04-30

**Authors:** Douglas F. Zatzick, Joan Russo, Doyanne Darnell, David A. Chambers, Lawrence Palinkas, Erik Van Eaton, Jin Wang, Leah M. Ingraham, Roxanne Guiney, Patrick Heagerty, Bryan Comstock, Lauren K. Whiteside, Gregory Jurkovich

**Affiliations:** 1 Department of Psychiatry & Behavioral Sciences, University of Washington, 325 Ninth Ave, Box 359911, Seattle, WA 98104 USA; 2 Division of Cancer Control and Population Sciences, National Cancer Institute, BG 9609 MSC 9760, 9609 Medical Center Drive, Bethesda, MD 20892-9760 USA; 3 School of Social Work, University of Southern California, Montgomery Ross Fisher Building, Room 339, Los Angeles, CA 90089 USA; 4 Department of Surgery, University of Washington, 325 Ninth Ave, Box 359796, Seattle, WA 98104 USA; 5 Harborview Injury Prevention Research Center, University of Washington, 325 Ninth Ave, Box 359960, Seattle, WA 98104 USA; 6 Department of Biostatistics, University of Washington, 1705 NE Pacific St, Box 357232, Seattle, WA 98195 USA; 7 Division of Emergency Medicine, University of Washington, 25 Ninth Ave, Box 359702, Seattle, WA 98104 USA; 8 Department of Surgery, University of California in Davis, 2221 Stockton Blvd, Cypress #3111, Sacramento, CA 95817 USA

**Keywords:** Traumatic injury, Multiple chronic conditions, Posttraumatic stress disorder, Depression, Suicidal ideation, Substance abuse, Effectiveness-implementation hybrid, Pragmatic clinical trial, American College of Surgeons, Policy

## Abstract

**Background:**

Each year in the USA, 1.5–2.5 million Americans are so severely injured that they require inpatient hospitalization. Multiple conditions including posttraumatic stress disorder (PTSD), alcohol and drug use problems, depression, and chronic medical conditions are endemic among physical trauma survivors with and without traumatic brain injuries.

**Methods/design:**

The trauma survivors outcomes and support (TSOS) effectiveness-implementation hybrid trial is designed to test the delivery of high-quality screening and intervention for PTSD and comorbidities across 24 US level I trauma center sites. The pragmatic trial aims to recruit 960 patients. The TSOS investigation employs a stepped wedge cluster randomized design in which sites are randomized sequentially to initiate the intervention. Patients identified by a 10-domain electronic health record screen as high risk for PTSD are formally assessed with the PTSD Checklist for study entry. Patients randomized to the intervention condition will receive stepped collaborative care, while patients randomized to the control condition will receive enhanced usual care. The intervention training begins with a 1-day on-site workshop in the collaborative care intervention core elements that include care management, medication, cognitive behavioral therapy, and motivational-interviewing elements targeting PTSD and comorbidity. The training is followed by site supervision from the study team. The investigation aims to determine if intervention patients demonstrate significant reductions in PTSD and depressive symptoms, suicidal ideation, alcohol consumption, and improvements in physical function when compared to control patients. The study uses implementation science conceptual frameworks to evaluate the uptake of the intervention model. At the completion of the pragmatic trial, results will be presented at an American College of Surgeons’ policy summit. Twenty-four representative US level I trauma centers have been selected for the study, and the protocol is being rolled out nationally.

**Discussion:**

The TSOS pragmatic trial simultaneously aims to establish the effectiveness of the collaborative care intervention targeting PTSD and comorbidity while also addressing sustainable implementation through American College of Surgeons’ regulatory policy. The TSOS effectiveness-implementation hybrid design highlights the importance of partnerships with professional societies that can provide regulatory mandates targeting enhanced health care system sustainability of pragmatic trial results.

**Trial registration:**

ClinicalTrials.gov NCT02655354. Registered 27 July 2015.

## Background

The overarching goal of the trauma survivors outcomes and support (TSOS) effectiveness-implementation hybrid clinical trial is to develop and implement a large scale, cluster randomized pragmatic demonstration project that directly informs national trauma care system policy targeting injured patients with presentations of posttraumatic stress disorder (PTSD) and related comorbidity. Physical injury occurs frequently in the USA and constitutes both a substantial source of individual suffering and a significant public health burden. Each year in the USA, over 30 million individuals present to acute care medical trauma center and emergency department settings for the treatment of traumatic physical injury [1–5]. Injured trauma survivors present to acute care medical settings after both intentional (e.g., gunshots, stabbings, physical assaults) and unintentional (natural disasters, motor vehicle crashes) injury events [6]. Annually, 1.5–2.5 million Americans are so severely injured that they require inpatient hospitalization [1–5]. Estimates suggest that approximately 1.5 million American youth and adults experience traumatic brain injury (TBI) annually [7, 8]. Physical injury with and without TBI constitutes a major public health problem for both civilian and veteran patient populations [9, 10]. Globally, traumatic injury accounts for approximately 16 % of the world’s burden of disease [11–13].

Multiple chronic conditions appear to be endemic among physical trauma survivors treated in US trauma care systems [14–16]. Recent commentary has explicated chronic conditions as conditions that last 1 year or more and require ongoing medical attention and/or limit activities of daily living [17–19]. Highly prevalent comorbidities include enduring PTSD, depression, and associated suicidal ideation, alcohol, and drug use problems, TBI, and chronic medical conditions such as hypertension, coronary artery disease, diabetes, and pulmonary disease [14, 20, 21].

Evidence-based, collaborative care intervention models for PTSD and related comorbidities exist [16, 22–25]. Collaborative care treatment models however, have yet to be broadly implemented throughout US trauma care systems; prior investigation by members of the interdisciplinary study team suggest that less than 10 % of US trauma centers routinely provide post-injury screening or integrated care management treatment targeting the cluster of PTSD and related comorbidities [26]. The enduring challenges presented by the chronic disease cluster of PTSD and comorbidities after injury require innovative research approaches that cut across the traditional domains of multiple NIH institutes (https://www.nihcollaboratory.org).

The investigation is designed as an effectiveness-implementation hybrid pragmatic trial that simultaneously aims to assess the treatment outcomes of the collaborative care intervention targeting PTSD and comorbidity, while also assessing the potential utility of the implementation strategy [27]. The study aims to determine if injured patients receiving the collaborative care intervention demonstrate significant reductions in PTSD symptoms when compared to control patients receiving care as usual. The study also aims to determine if intervention patients, when compared to control patients, will demonstrate significant reductions in depressive symptoms and associated suicidal ideation, alcohol use problems, and improvements in physical function.

Over the past decade, the study team has established a stakeholder partnership with the American College of Surgeons’ Committee on Trauma, whereby the results of pragmatic comparative effectiveness trials can be directly translated into policy mandates and best practice guidelines for the regulation of US trauma care systems [26, 28–31]. The investigative team will employ implementation science conceptual frameworks to better understand the potential uptake of the intervention model by trauma care systems nationwide.

### Implementation science and randomized clinical trial conceptual frameworks informing the TSOS trial

Recent commentary has noted a proliferation of models and conceptual frameworks that can potentially inform the design of investigations that target the widespread dissemination and implementation of health care interventions; in reviewing this literature, commentary suggests a systematic selection of optimal approaches for a particular investigation or health care delivery system [32–34].

**Fig. 1:**
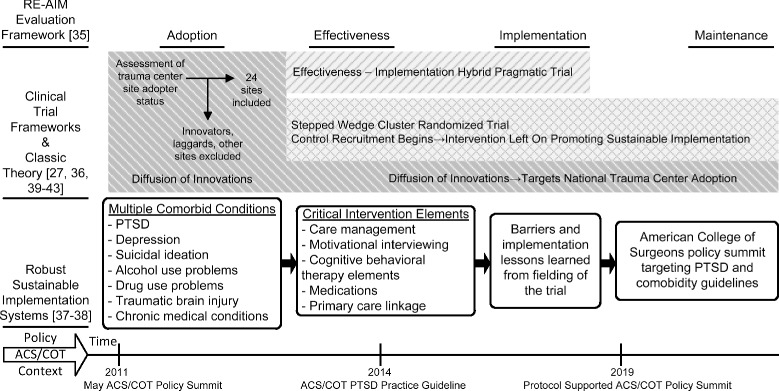
Implementation science conceptual framework informing the TSOS effectiveness-implementation hybrid pragmatic trial. *RE-AIM* reach effectiveness adoption implementation maintenance, *ACS/COT* American College of Surgeon’s Committee on Trauma, *PTSD* posttraumatic stress disorder

By necessity, multiple theoretical and applied perspectives inform the conceptual framework underlying the TSOS pragmatic trial design and implementation [32] (Fig. 1). The TSOS pragmatic trial design and implementation incorporates implementation science evaluation frameworks [35], and classic theories [36], as well as frameworks that address sustainable health care system change [37, 38]. The TSOS study is also informed by effectiveness-implementation hybrid design [27], pragmatic trials [39], and stepped wedge [40–43] clinical trial design considerations.

The implementation science conceptual frameworks influencing study design begin with the reach effectiveness adoption implementation maintenance (RE-AIM) evaluation framework that outlines clear stages of assessment for both effectiveness and implementation outcomes (Fig. 1). The RE-AIM framework provides a model for the integration of pragmatic trial results into routine trauma center practice [37]. Diffusion of innovation theory, which emphasizes the factors related to the intervention and setting characteristics, aids in the framing of the population-based sampling and adoption of trauma centers as well as descriptions of maintenance, based on the trial’s ability to target American College of Surgeons’ policy in order to shift “S-shaped” adopter curves nationally [36].

Clinical trial specific constructs and design features also contribute to the conceptual framework informing the TSOS study (Fig. 1). These include the emerging effectiveness-implementation hybrid design construct [27]. The TSOS trial simultaneously aims to determine the effectiveness of the stepped collaborative care intervention model in reducing PTSD symptoms and comorbid conditions, while also assessing the potential utility of the implementation strategy that uses American College of Surgeons’ policy to target regulatory mandates for trauma care systems nationally [37, 44].

The pragmatic-explanatory continuum indicator summary (PRECIS) pragmatic trial framework also informs the TSOS study [39]. Gold standards for pragmatic trial design and implementation include broad participant eligibility criteria, flexible intervention delivery, application by the full range of practitioners, and incorporation of rigorous prospective controls, preferably by randomization. Usual practice comparison conditions are frequently used in pragmatic trials [39, 44–48]. The optimal pragmatic trial is characterized by an intent-to-treat data analytic approach that includes all patients regardless of adherence [39]. The TSOS trial encompasses these pragmatic trial attributes by fielding a readily implementable collaborative care intervention that targets injured patients with the full spectrum of PTSD and related comorbidity with minimal exclusionary criteria.

Pragmatic trial process and outcome assessments have been conceptualized to be centrally measured, clinically meaningful, and require minimal adjudication [39, 44–48]. With regard to pragmatic trials in US trauma care systems, no one or even multiple administrative databases can be used to track outcomes among injured trauma survivors; thus for trauma care system pragmatic trials, scheduled telephone outcome assessments may by necessity occur as an addition to naturalistic follow-up. The PRECIS framework suggests that for some trials, outcome assessments must by necessity be obtained through contact with participants [39]. Similarly, the PRECIS framework takes into consideration the observation that in some trials that rely heavily on patient reported outcomes, some training in the assessment and adjudication may be desirable [39].

As an integrative model (Fig. 1), the Robust, Sustainable, Implementation Systems Framework [37] aids in combining the implementation science, pragmatic trial, and health care systems change conceptual frameworks that inform the TSOS study; conceptually the Robust, Sustainable, Implementation Systems Framework integrates elements of process and implementation models, determinant frameworks, and clinical trial frameworks (e.g., multiple comorbid condition targets and critical intervention elements) as well as the RE-AIM evaluation framework [32, 37] (Fig. 1). A further advantage of the framework is the flexible integration of recent work on barriers and facilitators of acute care medical screening and intervention guideline implementation ([49–51]). Policy relevance that ultimately enhances clinical trial population impact is also relevant to the Robust, Sustainable, Implementation Systems Framework [37, 52, 53]).

## Methods/design

### Design overview

The TSOS trial aims to recruit 960 patients, 40 at each trauma center site. The TSOS investigation employs a stepped wedge cluster randomized design in which sites are randomized sequentially to initiate the intervention. Patients are assessed at baseline in the emergency department or as trauma inpatients and again 3, 6, and 12 months after the injury. All sites have worked with the study team to implement an electronic health record (EHR) initial PTSD risk evaluation. Patients identified by the EHR evaluation as high risk for PTSD are formally assessed with the PTSD Checklist for study entry. Patients in the control condition will receive enhanced trauma center care as usual. Patients in the intervention condition will receive a stepped collaborative care intervention targeting PTSD and related comorbidities. The intervention begins with a 1-day workshop training in the collaborative care intervention core elements that include care management, medication, cognitive behavioral therapy (CBT), and motivational interviewing targeting PTSD and comorbidity. After the 1-day workshop, the site will receive supervision from the study team. Outcome analyses will incorporate both effectiveness and implementation spectrum assessments.

### Injury cohort definition, exclusions, and PTSD risk screening

Prior to the initiation of recruitment for the TSOS study, the investigative team worked with each trauma center site to define injury cohorts, characterize inclusion and exclusion phenotypes within the EHR [54], and implement the 10 domain EHR PTSD risk screen [16, 55]. The procedures used to define injury cohorts and characterize potential emergency department and trauma inpatient subjects for the recruitment process varied across sites depending on the capacity of individual sites to automate the screening procedure within or external to the EHR [56]. The automated form of the evaluation can be performed using EHR data queries or scheduled reports, while the manual form of the abstraction procedure involves reviewing individual health records; many sites have combined automated and manual procedures into a partially automated (i.e., hybrid) 10-domain risk screen.

**Fig. 2:**
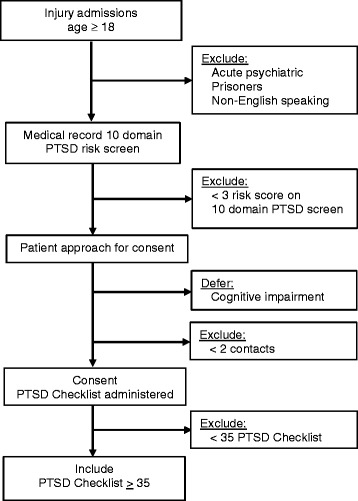
Patient flow through protocol. *PTSD* posttraumatic stress disorder. *PTSD* Checklist Civilian Version [58]

Injured patients of both genders over the age of 18 are included in the trial (Fig. 2). Prisoners and non-English speaking patients, will be excluded. Patients whose index injury was self-inflicted or are psychotic will receive immediate psychiatric treatment and will also be excluded from the trial. In order to assure adequate follow-up rates, patients must be able to provide two pieces of follow-up contact information.

Patients identified by EHR evaluation as at-risk for high early PTSD symptom levels with a score of >3 risk domains positive will then be formally screened for study entry with the PTSD Checklist Civilian Version [57, 58]. Patients scoring >35 on the PTSD Checklist will be followed longitudinally in the clinical trial portion of the investigation.

### Randomization

**Fig. 3:**
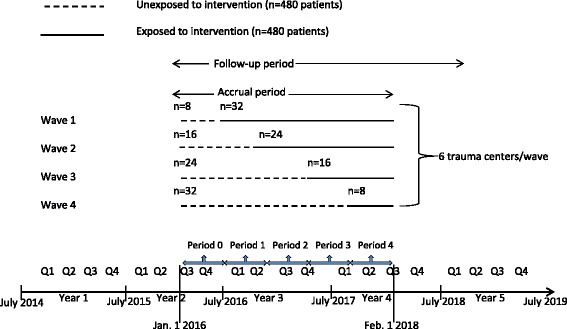
Stepped wedge cluster randomized trial design and timeline

Prior to initiation of patient recruitment, each of the 24 sites was randomized to one of four waves in the stepped wedge design. Each wave was assigned a specific proportion of control and intervention patient recruitment (Fig. 3). The study biostatistician randomized sites to waves using a computer generated algorithm. All interviewers conducting follow-up assessments will be blinded to patient intervention and control group status.

### Enhanced usual care control condition

The control patient subjects will receive enhanced usual trauma center care. Prior investigation suggests that usual posttraumatic care includes routine surgical, primary care, and emergency department visits, as well as the occasional use of specialty mental health services. The enhanced aspect of the usual care will consist of the recruiting provider informing the ward nurse currently covering the patient subject’s care of any distress they are experiencing as identified by a PTSD Checklist score of >35 or Patient Health Questionnaire (PHQ-9) item 9 > 1 indicating suicidal ideation, administered during the baseline interview.

### Stepped collaborative care intervention [16, 22–25]

Collaborative care treatment models that combine effective intervention elements and incorporate IT innovations have the potential to be flexibly implemented in order to prevent the development of the chronic condition cluster that includes PTSD and related comorbidity; collaborative care treatment models may also be effective in mitigating the impact of the acute injury event on symptom exacerbations in the large subpopulation of injury survivors who already carry a substantial pre-injury burden of chronic medical and other conditions [22, 25, 59–63] (Table 1).

**Table 1:** Core elements of collaborative care intervention targeting PTSD and comorbidity after injury

Essential element	Which disorders targeted	MCC strategic framework goals addressed^a^
Population-based EHR PTSD and comorbidity risk prediction	PTSD, depression, suicidal ideation, alcohol and drug use problems, TBI and chronic medical conditions after acute injury	Goal 1 objective D implement and efficiently use health information technology; EHR screening efficiently identifies constellation of PTSD and comorbidity in injured populations
Care management with trauma center to primary care linkage	Coordination of acute injury mental health and pre-existing chronic medical condition care	Goal 2 facilitate use of community based services and self-care management
Early post-injury medication history, reconciliation, and care coordination	PTSD, depression, pain, and TBI symptoms prevention. Chronic medical condition reconciliation and coordination	Goal 1 objective E prevent occurrence of new chronic conditions and mitigate the consequences of existing conditions
		Goal 2 objective C provide tools for medication management
Evidence-based MI embedded within care management	Targets alcohol and drug use problems and enhanced patient engagement	Goal 1 objective E prevent occurrence of new chronic conditions and mitigate the consequences of existing conditions
Evidence-based CBT embedded within care management	Targets PTSD, depression, pain, and TBI symptoms. Also targets enhanced patient self-efficacy	Goal 1 objective E prevent occurrence of new chronic conditions and mitigate the consequences of existing conditions
		Goal 2 objective A facilitate self-care management
Patient and caregiver-centered posttraumatic concern elicitation and improvement	Patient-centered concerns elicitation and improvement targets patient and family engagement in care of full MCC constellation	Goal 2 optimize self-care management and coordinated use of services by patient and caregivers
Caseload supervision and stepped measurement-based care implementation	PTSD, depression and associated suicidal ideation, alcohol and drug use problems, chronic medical conditions and acute physical injury	Goal 3 provide better information and education on treatment of MCCs to health care workers

*MCC* multiple chronic condition, *EHR* electronic health record, *PTSD* posttraumatic stress disorder, *TBI* traumatic brain injury, *MI* motivational interviewing, *CBT* cognitive behavioral therapy

^a^All study elements address MCC Goal 4 of Enhancing Research Knowledge on MCCs [17–19]

A large body of research has established the effectiveness of integrated care delivery models such as collaborative care in reducing depressive, anxiety, pain, and other somatic symptom presentations in conjunction with comorbid medical conditions in primary care settings [22, 23, 25, 61, 64–78]. Collaborative care treatments bring together effective medication and psychotherapeutic intervention elements with care management strategies that target reductions in care fragmentation and enhanced care coordination for patients with multiple chronic conditions (Table 1). A series of single site acute care medical studies now support the effectiveness of collaborative care models in targeting the PTSD and comorbidity chronic condition cluster [16, 22–25].

Study staff will visit the trauma center sites in order to perform a 1-day intervention workshop training. The workshop will provide an overview of the core elements of the PTSD and comorbidity intervention (Table 1). The trainers will review the intervention elements including care management, medications, motivational interviewing (MI) and CBT elements, and community linkage. Specific intervention procedures have been detailed previously [16, 22–25].

After the 1-day workshop training, the study team will initiate regular site care management supervisory calls in which the site interventionists will present cases to the supervisory team [16, 79]. These sessions will include coaching in concern elicitation, CBT, and MI elements embedded within care management, as well as problem-solving barriers to screening and intervention implementation for PTSD and related comorbidity. These calls will also include coaching on evidence-based medication prescription and supervisory team written feedback. The care managers will be able to contact MD and PhD study team members on a 24-h study cell phone or study assistance email should more urgent questions arise. While final patient subject follow-up interviews take place approximately 12 months post-consent, intervention activities are anticipated to conclude approximately 6 months after patient subjects consent into the trial. During the final months of treatment, the interventionist will discuss with the patient strategies for maintaining treatment gains. This means proper handoff of medication prescription management to a patient subject’s preferred primary care or other medical provider, linkage to community resources, engaging family and community support, and when indicated psychotherapy referrals.

### Assessments

The TSOS assessment approach incorporates both effectiveness and implementation outcome evaluations [80]. The timing and content of the TSOS outcome assessments are delineated in Tables 2 and 3. The primary effectiveness evaluations are patient-reported outcome measures that include assessments of the study primary and secondary outcomes (Table 2). The RE-AIM evaluation framework informs the implementation outcome assessments [35] (Table 3). Selected outcome assessments are described in further detail below.

A. Primary study patient-reported outcome: PTSD symptom assessment [58, 81]
  The PTSD Checklist is a 17-item self-report questionnaire that will be used to assess PTSD symptoms. A series of investigations have demonstrated the reliability and validity of the PTSD Checklist across trauma-exposed populations. PTSD Checklist scores of >35 in the days and weeks after injury admission have been shown to be associated with the development of higher PTSD symptom levels over the course of the year after injury [55].
B. Secondary study patient-reported outcomes: depressive symptoms, suicidal ideation, alcohol use problems, and physical function
*Depressive symptoms*. The 9-item Patient Health Questionnaire (PHQ-9) brief depression severity measure will be used to assess depressive symptoms [82]. The PHQ-9 has established reliability and validity in acute and primary care medical patients [16, 25, 83].
*Suicidal ideation*. The PHQ-9, item 9, will be used to assess for suicidal ideation [84].
*Alcohol use problems*. The Alcohol Use Disorder Identification Test (AUDIT), a ten-item screening instrument for the early identification of problem drinkers will be used to assess alcohol use problems before and after the injury hospitalization [85]. The AUDIT’s reliability and validity are well established, and the scale has been widely used in acute and primary care medical settings [16, 25, 85–87].
*Limitations in physical function*. The investigation will use the Medical Outcomes Study Short Form (MOS SF) SF-12 at baseline and SF-36 at 3-, 6-, and 12-month follow-up to assess physical, role, and social functional outcomes. The SF-12/36 has established reliability and validity [88], and the measure has been used extensively with traumatically injured populations [89–91].
C. Baseline patient trauma center/emergency department electronic health record (EHR) assessment
  EHR data will be collected from each of the 24 sites during the recruitment of study patients. Similarly, trauma registry data will be obtained from each of the 24 sites that will contain EHR derived international classification of diseases (ICD) codes and other clinical data.
*EHR 10 item PTSD risk factor screen*. A previously developed EHR screen will be used to assess admitted injured trauma survivors at risk for the development of PTSD [55]. The screen utilized ten data elements that are both associated with increased risk for PTSD and that are readily available in any robust EHR system. When the ten data elements were used to predict scores on the PTSD Checklist of >35, the EHR screen demonstrated adequate sensitivity (0.71), specificity (0.66), and area under the ROC curve (0.72) [55].
*Injury severity*. Injury severity will be abstracted from the medical record using a conversion software program that transforms recognized ICD codes into the Abbreviated Injury Scale (AIS) and subsequent injury severity scores (ISS) [92].
*Traumatic brain injury (TBI)*. Mild, moderate, and severe TBI will be identified and categorized from electronic record abstracted ICD codes indicative of traumatic injury.
*Medical conditions*. Comorbid chronic medical conditions will also be taken from medical record and trauma registry data and will be derived from ICD diagnostic codes [93, 94]. Chronic medical comorbidity will also be assessed through patient self-report during the follow-up interviews.
D. Provider assessments
*Trauma center organizational assessments* [95–100]. The study will modify previously developed organizational culture and climate assessment scales to evaluate trauma center organizational characteristics related to PTSD and comorbidity service implementation [95, 101, 102]. Organizational implementation scales will assess the extent to which trauma centers were able to adapt to the changes required by PTSD and comorbidity screening and intervention service development [96, 101]. Trauma center provider attrition from the study and turnover will also be examined. Following the procedure established in the study team’s previous Disseminating Organizational Screening and Brief Intervention Services (DO-SBIS) pragmatic trial, ten providers from each of the 24 sites will be identified through an organizational mapping procedure to be part of the organizational work unit impacted by screening and intervention service delivery [101, 103]. These ten providers will complete the organizational assessment prior to beginning intervention activities and again in study year 4 after all patient intervention is complete.
*Trauma center provider exposure to critical incidents and job stress* [104, 105]. Previously developed items will be used to assess trauma center provider job-related stress (e.g., call frequency, work volume) [104]. Provider secondary traumatic stress, lifetime trauma, and PTSD symptoms will also be assessed [6, 58, 81, 106, 107].
*Intervention provider standardized patient assessments* [108]. In the study team’s prior DO-SBIS pragmatic trial focusing on alcohol screening and intervention, standardized patient fidelity assessments were used to assess fidelity to MI interventions delivered by front-line trauma center providers. Each standardized patient interview was scored using the Motivational Interviewing Treatment Integrity (MITI) coding system. The MITI will again be used to code patient standardized interviews in the current TSOS study.
E. Exploratory health economic evaluation
  The cost assessments are intended to contribute to an understanding of the resource implications of the intervention and to American College of Surgeons’ and other national policy dialogues of post-injury health service utilization and costs to support subsequent intervention scale-up and spread [109–118]. The investigation will collect detailed information on the following: (1) the costs of intervention implementation and delivery, (2) post-injury health service utilization costs (e.g., inpatient, skilled nursing facility, emergency room, and outpatient utilization), and (3) the costs of patient medications post-injury. Costs of intervention are likely to be dwarfed by the total costs of post-injury care, which would make it difficult to estimate the incremental costs of intervention precisely, given our sample size. The health care resource utilization and cost analyses constitute an important exploratory aim of the investigation.
F. Study team logging procedures
  The approach to trial logging simultaneously aimed to satisfy the pragmatic trial requisite for the minimization of time intensive research methods that require extensive adjudication [39] and the implementation science goal of understanding and documentation of trial processes that could yield sustainable maintenance of screening and intervention procedures [27].
  Because pragmatic trials tacitly aim to provide health care delivery settings with readily implementable intervention models, logging procedures that differentiate study team activities related to (1) the fielding of the trial, (2) the implementation of evidence-based interventions, (3) costing and economic analyses, and (4) regulatory procedures may be critical for pragmatic trial design and implementation. Previously articulated procedures for the logging of clinical trial and implementation activities were adapted for the current pragmatic trial approach [119, 120]. A pragmatic trial framework that emphasized time efficiency and minimal adjudication of logged activities argued for optimizing parsimony in the logging approach [39]. All study research team site contacts, including email, phone, and in-person site contacts, and all study team consultant (e.g., trauma surgery champion) contact with sites are logged. Both 24 site specific logs and domain specific logs (i.e., trial specific activities, evidence-based intervention implementation, sustainability, and economic considerations) will be maintained. As part of the study’s mixed method assessment procedures, the logs and field notes will be reviewed on an approximately monthly basis with the investigation’s mixed methods consultant [70, 121–123].
G. Semi-structured provider interviews [124, 125]
  After the completion of recruitment and intervention activities, semi-structured interviews will be conducted with interventionists from each of the 24 trauma center sites. The interviews will explore barriers and facilitators of implementation of screening, intervention, and quality documentation procedures for PTSD and comorbidity at trauma center sites. The interviews will also explore the potential sustainability of study procedures.

**Table 2:** Effectiveness assessments and timing of administration

Study measure	Baseline	3 months	6 months	12 months
EHR 10 item PTSD evaluation [55]	X			
ICD injury severity	X			
ICD TBI severity	X			
ICD/self-report chronic medical conditions	X	X		
EHR and self-reported demographics	X			
Consciousness/Glasgow Coma Scale [144, 145]	X			
PTSD (PTSD Checklist DSM-IV & DSM-5) [58, 81]	X	X	X	X
Depression (PHQ-9) [82]	X	X	X	X
Suicidal ideation (PHQ-9 item 9) [82, 84]				
Alcohol (AUDIT) [85]	X	X	X	X
Illegal and prescription drug use (DAST) [146]	X	X	X	X
Pain (Brief Pain Inventory) [147, 148]	X	X	X	X
Postconcussive symptoms [90, 149, 150]	X	X	X	X
Functioning (MOS SF12/36) [88]	X	X	X	X
Violence risk behaviors [24]	X	X	X	X
Pre-injury trauma [6, 106, 107]		X		
Recurrent traumatic events [6, 106, 107]			X	X
Reactions to research participation [25]	X	X	X	X
Satisfaction with care [16, 25]	X	X	X	X
Health services, work and cost [14, 151–154]	X	X	X	X
Medication use [14, 16, 25, 151]	X	X	X	X
EHR/trauma registry data [14, 151]	Ongoing automated data			

*EHR* electronic health record, *PTSD* posttraumatic stress disorder, *ICD* international classification of diseases, *DSM* Diagnostic and Statistical Manual of Mental Disorders, *PHQ-9* Patient Health Questionnaire, *AUDIT* Alcohol Use Disorders Identification Test, *DAST* Drug Abuse Screen Test, *MOS SF* Medical Outcomes Study Short Form

**Table 3:** TSOS implementation assessments and corresponding RE-AIM framework domains

Assessment	Patient, provider or site assessment	How assessed	*N*	RE-AIM domain, level
Characteristics of 24 study sites versus all other US sites [35]	Site recruitment	CONSORT	24/224	Adoption, site
Organizational change, climate and culture surveys [95–102]	Trauma center providers	Web-based survey	10*24	Implementation, provider
Weekly recruitment log activity [16, 25, 70]	PTSD interventionist	Recruiting logs	24	Implementation, provider and site
Clinical notes in decision support tool [16]	PTSD interventionist	Decision support tool	24	Implementation, provider
Patient flow through protocol utilizing trauma registry, recruitment data [16, 25, 70]	Patient flow	CONSORT	960	Reach, patient
PTSD and comorbidity, gender and ethnicity groups [16, 25, 70]	Patient outcomes	Telephone interview	960	Effectiveness, patient
EHR, trauma registry, self-report logs [16, 25, 70]	Patient outcomes	Multiple sources	960	Implementation, patient
>6 months follow-up after intervention [16, 25, 70]	Patient 12-month follow-up	Phone	960	Maintenance, patient
Semi-structured key informant interviews [121–125]	PTSD interventionist	Phone	24	Implementation and maintenance, provider and site
National trauma center questionnaire [26, 30]	All US level I centers	Web	224	Maintenance, site

*RE-AIM* reach effectiveness adoption implementation maintenance, *EHR* electronic health record

### Statistical analysis plan

#### Study aims and hypotheses

The primary hypothesis is that the intervention group when compared to the control group will demonstrate significant reductions in PTSD symptoms over the course of the year after injury. Secondary hypotheses are that intervention patients when compared to control patients will demonstrate significant reductions in depressive symptoms and associated suicidal ideation, significant reductions in alcohol use problems, and improved post-injury physical function.

All primary statistical analyses will be conducted using intent-to-treat methods. The primary goal of the statistical analyses is to examine and compare trends over time in the symptoms of PTSD. This analytic approach will be replicated for all secondary outcomes; secondary analyses will examine trends over time for depression, alcohol use, and physical function. The major outcome variables are the continuous and dichotomous assessments of PTSD (PTSD Checklist [81]), depression (PHQ-9 [82]), alcohol use problems (AUDIT [85]), and physical function (MOS SF-36 PCS [88, 126]).

The study team will use mixed effects regression models to test the hypotheses. The investigative group has extensive experience with these analytic approaches in the analyses of longitudinal data after injury. These analytic approaches allow for the modeling of longitudinal data on patients, nested within trauma center sites (see also sample size and power discussion below for a more in-depth explanation of clustering). An important potential advantage of using longitudinal mixed models is the ability to use partial data on those subjects with missing data, and therefore potentially ameliorate selection bias due to drop out. In addition, mixed models naturally structure patient and trauma center heterogeneity specifically allowing for random effects such as individual intercepts and slopes over time. Longitudinal regression models also allow the use of baseline covariates that may be prognostic or reflective of the study design.

Exploratory analyses will assess the impact of the intervention on primary and secondary outcomes for patients with and without pre-injury chronic medical conditions and those with and without TBI. Exploratory analyses will also assess for significant reductions in suicidal ideation, pain, and drug use problems in intervention patients when compared to control patients.

The study team will use a stepped wedge cluster randomized design for the TSOS protocol [40–43] (Fig. 3). Variability in multiple trauma center characteristics can impact rates of recruitment (e.g., admission volumes, EHR capacity), rates of PTSD (e.g., percentages of patients with violent injury admissions, intensive care unit admission rates), and the ability to follow patients longitudinally (e.g., patient demographic characteristics such as being homeless, clinical characteristics such as substance use problems). The stepped wedge design randomizes level I trauma center sites to sequentially initiate the intervention, thus allowing within site pre- and post-intervention comparisons, as well as between site comparisons. An additional advantage of the stepped wedge design for the protocol is that it would be impractical to roll out the entire intervention at 24 sites simultaneously. Finally, from an implementation science perspective, there is an advantage to having the intervention ongoing at the end of the study at every site, should the intervention demonstrate a significant impact on PTSD and comorbidity (Fig. 1). Given that there is little threat of contamination at each site across intervention and control patients and that the UH3 can accommodate the increased potential length of active recruitment and follow-up, the stepped wedge design appears to be an optimal choice for the TSOS protocol.

### Sample size and power

A number of issues specific to the design and analyses of cluster randomized trials are addressed by the current power analyses [127–131]. A key consideration for the trial is the nesting of patients within trauma center sites and the ascertainment of associated intraclass correlations (ICC). The study team has extensive experience with prior multisite trauma center observational and pragmatic clinical trial investigations. Sample size estimates were therefore adjusted for the clustering of patients within trauma center sites, using appropriate ICCs derived from the study team’s prior multisite investigations (Table 4).

**Table 4:** Stepped wedge power for TSOS outcomes

Continuous outcomes	PTSD Checklist	PHQ-9	AUDIT	MOS SF-PCS
Cluster size at baseline	40	40	40	40
Cluster size estimation at 12-month (25 % attrition)	30	30	30	30
Total number of clusters	24	24	24	24
Alpha	0.05	0.05	0.05	0.05
Power	0.8	0.8	0.8	0.8
ICC	0.02	0.0259	0.02	0.02
Baseline mean (SD)	50 (15)	14 (6)	10 (5)	50 (10)
Autocorrelation	0.7	0.7	0.7	0.7
Follow-up time points (including baseline)	4	4	4	4
Minimal detectable effect size	0.23	0.23	0.23	0.23

*PTSD* posttraumatic stress disorder, PTSD Checklist Civilian Version [58], *PHQ-9* Patient Health Questionnaire [82], *AUDIT* Alcohol Use Disorders Identification Test [85], *MOS SF PCS* Medical Outcomes Study Short Form Physical Components Summary Score [126], *ICC* intraclass correlation, *SD* standard deviation

Some attrition is expected in the study sample due to the research context and the population under study (i.e., low income, ethnoculturally diverse, injured trauma survivors). Prior studies by the investigative group have consistently achieved follow-up completion rates >75–80 % at 6–12 months post-injury with this population [16, 24, 25, 132]. Estimates derived from these rates are incorporated into the descriptions of subject flow and power analyses. Table 4 delineates the parameters used to estimate power for the PTSD Checklist, PHQ-9, AUDIT, and MOS SF-PCS. Sample size estimates were derived using the STATA statistical package [133]. With each of the 24 trauma center sites recruiting 40 patients into the study, the study has 80 % power to detect effect sizes of 0.23. These effect sizes are smaller than our previously observed treatment effect for PTSD symptoms of 0.34. In prior investigations, PTSD treatment effects of 0.34 have been associated with clinically significant and policy relevant functional outcome improvements [25].

### Mixed method analysis

Mixed methods will be used to integrate the findings from the key informant interviews with pragmatic trial results. The design taxonomy follows a sequential (QUAN → qual) structure in which qualitative data collected from key informants will be used to explain quantitative data results from the pragmatic trial [134, 135]. Qualitative data will therefore be used to expand upon the results of the pragmatic trial in order to understand the implementation and policy processes as experienced by key stakeholders. Second, the sequential QUAN → qual mixed methods design will be used to provide an understanding of pragmatic trial results that require further explanation (e.g., control patients that demonstrate substantial improvement in outcomes, despite not receiving intervention). Results of the mixed method analyses will be presented through a number of modalities that may include key informant narratives, tabular representation of themes with illustrative quotes, and thematic counts [136–139].

### Trial status

**Fig. 4:**
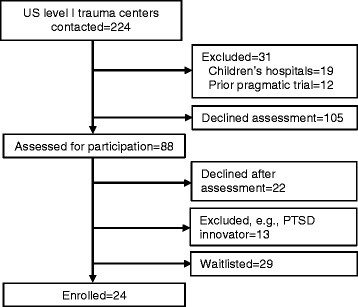
Site recruitment consort. *PTSD* posttraumatic stress disorder

Over the course of the pre-recruitment phase, the TSOS study team has enrolled the 24 trauma center sites that will participate in the trial. The goal of the selection process was to recruit 24 level I trauma centers nationally that would be capable of efficiently implementing the study procedures. The study team sent notification emails and/or contacted by telephone individuals at all US level I trauma centers (Fig. 4). Responding centers were asked questions about current PTSD screening and intervention practices; the study excluded the less than 10 % of “innovator” sites nationally that were already routinely screening and intervening for PTSD and related comorbidity [26]. Pediatric specialty trauma centers were also excluded from the investigation, as elements of the intervention (e.g., the administration of psychopharmacological agents targeting PTSD) are less well established for patients under the age of 18.

With the exception of pediatric trauma center specialty status, the organizational characteristics of the 24 participating sites does not substantially differ from the characteristics of all US level I trauma centers potentially eligible for the study (Table 5). Broad adoption and site level generalizability is an important aim of the investigation as it targets American College of Surgeons’ policy for PTSD and comorbidity screening and intervention for all trauma centers nationwide.

**Table 5:** Organizational characteristics of TSOS study versus other US level I trauma centers (*N* = 222)^a^

Characteristic	TSOS TC *n* = 24 *n* (%)	Other TCs *n* = 198 *n* (%)	*P*
American College of Surgeons accredited	17 (70.8)	74 (37.4)	0.01
Region of country			0.40
Midwest	7 (29.2)	64 (32.3)	
South/Southeast	4 (16.7)	30 (15.2)	
Northeast/East	5 (20.8)	63 (31.8)	
West	4 (16.7)	28 (14.1)	
Central	4 (16.7)	13 (6.6)	
Rural status	3 (12.5)	24 (12.1)	1.0
Population served			0.03
Adult	7 (29.2)	92 (46.5)	
Adult and pediatrics	17 (70.8)	82 (41.4)	
Pediatrics	0 (0.0)	23 (11.6)	
Missing	0 (0.0)	1 (0.5)	
Teaching hospital	23 (95.8)	162 (81.8)	0.14
Council of teaching hospitals	22 (91.7)	143 (72.2)	0.04
University affiliation	24 (100.0)	189 (95.5)	0.60
Median (IQR)			
Number of interns/residents	327 (282)	224 (297)	0.11
Number of hospital beds	575 (296)	534 (318)	0.40
Number of inpatient admits	26,971 (16,311)	25,699 (14,978)	0.28

*TC* trauma center, *IQR* interquartile range

^a^Two of 224 sites were missing organizational data

## Discussion

The current effectiveness-implementation hybrid is innovative in its combination of pragmatic trial and implementation science conceptual frameworks. The effectiveness-implementation trial is a “hybrid type II” design that uses a novel, yet time-tested, American College of Surgeons’ policy mechanism as a targeted implementation strategy [27]. Curran and colleagues note that to enhance the relevance of pragmatic studies, comparative effectiveness trials may require modification in order to have increased policy relevance [27]. Curran and colleagues also critique pragmatic comparative effectiveness studies for exclusively targeting effectiveness outcomes with little attention to the implementation processes relevant to general practice settings; these authors note that in contrast, implementation trials focus on the uptake and adoption of clinical interventions by providers and systems of care [27].

As part of the study’s emphasis on implementation, an American College of Surgeons’ policy summit is scheduled in the final years of the trial. The aim of the policy summit is to facilitate rapid translation of trial results into national policy. The College oversees the development of national policy mandates and clinical best practice guidelines that inform the integrated operation of US trauma centers and affiliated trauma care systems [28, 29, 140]. The College has successfully linked trauma center funding to verification site visits and other quality indicators [28, 141, 142].

In January of 2005, the College made a landmark policy decision to mandate health services targeting screening and intervention for alcohol-related disorders as a requisite for trauma center accreditation [28]. Prior pragmatic randomized clinical trial investigations from the study team provided evidence supporting the College’s alcohol mandate [22, 30, 143]. In May of 2011, the investigators presented results from effective, NIH funded, PTSD screening and intervention trials at a College policy summit [22–24, 70]. For the first time, the College has included PTSD screening and intervention as a best practice level recommendation in national guidelines for trauma center care. These new College clinical guidelines set the stage for the current effectiveness-implementation hybrid trial that tests high quality, feasibly implemented, screening and intervention procedures for PTSD and related comorbidity. Simultaneously, as the investigation is being conducted, the study team will be actively developing a policy agenda targeting the use of pragmatic trial results to directly inform the policy discussion in the final years of the grant.

The potential for a policy target sets up a novel staged implementation context whereby the fielding of the trial and the implementation of the evidence-based intervention can yield insight into the sustainable delivery of PTSD screening and intervention procedures for trauma centers nationwide. In this context, previously described Rapid Assessment Procedures that harness clinical ethnographic methods to embed participant observation within front-line implementation teams have great potential utility [70, 121–123]. These methods rely on the study team collection of implementation logs and field notes; these logs and field notes can be productively reviewed on a regular basis with the study mixed method consultant in order to maximally harness field observations. This Rapid Assessment Procedures approach simultaneously satisfies the pragmatic trial requisite for minimization of time intensive research methods that require extensive adjudication and the implementation science goal of understanding and documentation of trial processes that could yield sustainable maintenance of screening and intervention procedures.

In summary, a hybrid effectiveness-implementation spectrum pragmatic trial targeting screening and intervention for PTSD and comorbidity can be readily designed and feasibly implemented across US level I trauma centers. These findings highlight the importance of partnerships with professional societies such as the American College of Surgeons’ that can provide regulatory mandates in order to enhance widespread implementation of pragmatic trials results.

## Abbreviations

CBT: cognitive behavioral therapy
DO-SBIS: disseminating organizational screening and brief intervention services
DSM: diagnostic and statistical manual
EHR: electronic health record
ICC: intraclass correlation
ICD: international classification of diseases
MI: motivational interviewing
PRECIS: pragmatic-explanatory continuum indicator summary
PTSD: posttraumatic stress disorder
RE-AIM: reach effectiveness adoption implementation maintenance
SD: standard deviations
TBI: traumatic brain injury
TSOS: trauma survivors outcomes and support
